# Electrophysiological Properties of Substantia Gelatinosa Neurons in the Preparation of a Slice of Middle-Aged Rat Spinal Cord

**DOI:** 10.3389/fnagi.2021.640265

**Published:** 2021-03-10

**Authors:** Yang Li, Shanchu Su, Jiaqi Yu, Minjing Peng, Shengjun Wan, Changbin Ke

**Affiliations:** Hubei Key Laboratory of Embryonic Stem Cell Research, Department of Anesthesiology, Institute of Anesthesiology & Pain (IAP), Taihe Hospital, Hubei University of Medicine, Shiyan, China

**Keywords:** spinal cord slice, middle-aged rodent, patch-clamp, action potential, synaptic transmission

## Abstract

A patch-clamp recording in slices generated from the brain or the spinal cord has facilitated the exploration of neuronal circuits and the molecular mechanisms underlying neurological disorders. However, the rodents that are used to generate the spinal cord slices in previous studies involving a patch-clamp recording have been limited to those in the juvenile or adolescent stage. Here, we applied an N-methyl-D-glucamine HCl (NMDG-HCl) solution that enabled the patch-clamp recordings to be performed on the superficial dorsal horn neurons in the slices derived from middle-aged rats. The success rate of stable recordings from substantia gelatinosa (SG) neurons was 34.6% (90/260). When stimulated with long current pulses, 43.3% (39/90) of the neurons presented a tonic-firing pattern, which was considered to represent γ-aminobutyric acid-ergic (GABAergic) signals. Presumptive glutamatergic neurons presented 38.9% (35/90) delayed and 8.3% (7/90) single-spike patterns. The intrinsic membrane properties of both the neuron types were similar but delayed (glutamatergic) neurons appeared to be more excitable as indicated by the decreased latency and rheobase values of the action potential compared with those of tonic (GABAergic) neurons. Furthermore, the glutamatergic neurons were integrated, which receive more excitatory synaptic transmission. We demonstrated that the NMDG-HCl cutting solution could be used to prepare the spinal cord slices of middle-aged rodents for the patch-clamp recording. In combination with other techniques, this preparation method might permit the further study of the functions of the spinal cord in the pathological processes that occur in aging-associated diseases.

## Introduction

Whole-cell patch-clamp recordings of the spinal cord slices represent an essential experimental system in neuroscience, which has enabled detailed studies of the neuronal architecture, spinal cord circuits, and their functions in the processes that are associated with both the physiological and pathological conditions (Konnerth, 1990). Several studies have been carried out to classify the γ-aminobutyric acid-ergic (GABAergic) and glutamatergic neurons in the lamina II layer [the substantia gelatinosa (SG)] of the spinal dorsal horn into several types according to morphology and electrophysiological properties (Maxwell et al., 2007; Yasaka et al., 2010). In addition, several researchers have studied primary afferent axonal projections and local neuronal circuits in the spinal cord (Brumovsky et al., 2006; Uta et al., 2010). These cellular and synaptic features of SG neurons are typically performed in the spinal cord slices obtained from newborn or young rodents; however, the features of the spinal cord neurons of middle-aged and older rodents remain unknown. In recent years, the contributions of the spinal cord have been reported in arthritis (Park et al., 2016), Parkinson's disease (Charles et al., 2018), diabetic neuropathy (Inam-U-Llah et al., 2019), Alzheimer's disease (Yuan et al., 2019a), and chronic pain (Bardoni et al., 2019). Epidemiological surveys suggest that middle-aged and older people are more vulnerable to these diseases. Unfortunately, since the current spinal cord slice preparation procedures are not suitable for aging rodents, the electrophysiological properties of SG neurons have not been evaluated by the *in vitro* recordings from the animal models of these diseases.

Some successful attempts have been made to perform a patch-clamp recording in the brain slices that are obtained from middle-aged and older rodents (Ting et al., 2014, 2018). Based on these pioneering works, the N-methyl-D-glucamine (NMDG) protective recovery method has been widely adopted by numerous published studies in the preparation of adult rodent brain slice. These acute brain slice studies have targeted several brain regions, such as the neocortex (Jin et al., 2019), the hippocampus (Chen et al., 2020; Takahashi et al., 2020), the striatum (Kummer et al., 2015), the thalamus (Zhu et al., 2019b), and the hypothalamus (Yuan et al., 2019b). The abovementioned method is well-suited for the recording of neuronal firing and synaptic transmission in the brain slices derived from transgenic or *in vivo* viral-transduced animals and has been used to perform functional Ca^2+^ imaging for evaluating the neuronal activity. However, it remains unknown whether this method can be used to prepare the spinal cord slices from middle-aged rodents in patch-clamp recording experiments.

In the present study, we applied this method for the preparation of the spinal cord slices from middle-aged rats and attempted to perform the patch-clamp recordings of SG neurons. According to the observed action potential firing patterns, we were able to classify the neurons as either delayed (glutamatergic) or tonic (GABAergic) subtypes and analyze synaptic transmission characteristics. In future studies, the combination of this technique, along with transgenic, chemical genetic, optogenetic, pharmacological, and behavioral methodologies, will offer a more comprehensive approach to study the pathological processes that occur in the spinal cord in aging-associated diseases.

## Materials and Methods

### Animals

Female Sprague Dawley rats were obtained from the Institute of Laboratory Animal Science, Hubei University of Medicine, China. All animals were individually housed in plastic cages with a 12-h light/dark cycle. For these experiments, 10-month-old rats (300–350 g) were used, and the experimental protocol was approved by the Animal Care and Use Committee of the Hubei University of Medicine, in agreement with the National Institutes of Health Guide for the Care and Use of Laboratory Animals.

### Solution Preparation

To prepare 1 M CaCl_2_, 1.11 g CaCl_2_ was dissolved in 10 ml ultrapure water and filtered through a 0.22-μm filter. Aliquots were divided into 1-ml centrifuge tubes and stored at −20°C until use. This solution was thawed for use in the preparation of both the NMDG-HCl artificial cerebral spinal fluid (aCSF) and normal aCSF.

N-methyl-D-glucamine HCl aCSF (93 mM NMDG, 93 mM HCl, 2.5 mM KCl, 1.25 mM NaH_2_PO_4_, 30 mM NaHCO_3_, 20 mM HEPES, 15 mM glucose, 0.5 mM CaCl_2_, 10 mM MgSO_4_, 2 mM thiourea, 5 mM sodium ascorbate, 3 mM sodium pyruvate, and 12 mM acetylcysteine) was titrated to pH 7.3–7.4 with HCl, and the osmolality was adjusted to 320 mOsmol/kg. A 250-ml glass beaker containing 200 ml of the resulting NMDG-HCL aCSF was prechilled on ice under a constant condition of 95% O_2_ and 5% CO_2_ for 30 min. Then, 150 ml of the chilled solution was transferred into a precooled flume base on a vibratome (Lecia, VT 1000s) while maintaining constant carbogenation. A volume of 25 ml NMDG-HCl was withdrawn into a syringe connected to a flexible pipe and a syringe needle for intracardiac perfusion. Another 10 ml of the prepared solution was placed into a glass dish, which was kept on ice for the immersion of the dissociated spinal cord. A second 250-ml glass beaker containing 150 ml NMDG-HCl aCSF was prepared under a condition of 32°C and constant carbogenation during initial slice recovery.

Normal aCSF (126 mM NaCl, 2.5 mM KCl, 1.25 mM NaH_2_PO_4_, 26 mM NaHCO_3_, 25 mM glucose, 5 mM HEPES, 2 mM CaCl_2_ 2H_2_O, and 2 mM MgSO_4_) was freshly prepared on the day of the experiment at room temperature with constant 95% O_2_ and 5% CO_2_.

Intracellular pipette solution (135 mM potassium gluconate, 2 mM MgCl_2_, 10 mM HEPES, 0.5 mM EGTA, 2 mM Mg-ATP, and 0.5 mM Na_3_-GTP) was adjusted to pH 7.4 with 0.5 M KOH, and the osmolality was adjusted to 310 mOsmol/kg with glucose. After filtration, aliquots were divided into 1-ml centrifuge tubes and stored at −20°C until use.

To prepare 2% agarose, 0.1 g of low-gelling-point agarose was dissolved in 5-ml phosphate-buffered saline (PBS) and heated in an oven. The solution was placed in a modified 10-ml syringe containing a 2-mm diameter metal bar and was allowed to solidify. After the solidification of the solution, the bar was removed to obtain agarose in the form of a cylindrical tube.

The agarose was acquired from Shanghai Bay Gene Biotechnologies Company (Shanghai, China). All other reagents were obtained from Sigma-Aldrich (St. Louis, Missouri, USA).

### Spinal Cord Slice Preparation

The rats were deeply anesthetized using pentobarbital (40 mg/kg, i.p.) and transcardially perfused with 25 ml ice-cold NMDG-HCl aCSF to induce rapid cooling of the body and retard metabolism. The skin was opened along the midline of the back using a scalpel, and the S1–T5 vertebral column was removed. The muscle was removed to expose the bone, and the intervertebral space was broken using two pairs of hemostatic forceps to expose the spinal cord. The spinal cord was removed and completely immersed in the ice-cold NMDG-HCl solution. Under a stereomicroscope (Olympus, SZX10), the cord was grasped using microsurgery forceps at the cervical end, and the roots were removed by pulling them from the cervical end toward the caudal end. Holding the caudal end of the spinal cord with forceps, the dorsal horn was inserted into the center hole of the cylindrical agarose tube. Then, both ends were removed, and the agarose block was attached to a specimen holder by using adhesive glue. The specimen holder was placed in the slicing machine (Lecia VT 1000), and proper alignment was verified.

The blade carrier was set to the desired speed and oscillation frequency, and the tissue was sliced in transverse sections at 300-μm increment until the lumbar enlargement was fully sectioned. Slices were collected with a polished glass Pasteur pipette and transferred into prewarmed (32°C) NMDG-HCl aCSF for 10 min. Slices were then transferred to normal aCSF and were allowed to recover for 30–60 min at room temperature.

### Patch-Clamp Recordings

A spinal slice immersed in aCSF was placed in a recording chamber and covered with a nylon mesh. A peristaltic pump was used to circulate oxygenated aCSF at 2–4 ml/min. The neurons were identified by using a differential interference contrast microscope (DIC; Lecia SP8) connected to a camera (DAGE-MTI, IR-1000E). The patch-clamp recording was performed at room temperature. Patch pipettes (3–4 MΩ) were prepared using a micropipette puller (P97, Sutter Instrument) and filled with an intracellular pipette solution. The pipette was placed in the aCSF by using a micromanipulator, and positive pressure (0.1 ml from a 1-ml syringe) was used to remove any dirt or debris from the vicinity of the micropipette. The pipette was manipulated until it was slightly attached to the neuron. Positive pressure was released, and gentle negative pressure was applied to assist the formation of a giga seal with the neuron. Then, a transient suction was applied and a good whole-cell configuration was created. During the recording period, the series resistance was monitored. Recordings were discarded if the series resistance reached more than 30 MΩ, a membrane resistance became < 0.8 GΩ, or leak currents became larger than 100 pA. The input resistance was measured as the slope of linearly fitted current–voltage plots after the application of −60–0 pA of hyperpolarizing current for 500 ms. To elicit an action potential, an increasing amount of depolarizing current was injected for 20 ms in a stepwise manner from 0 to 200 pA in 20-pA increment. A 500-ms, long-lasting, 50 pA current was administered to identify the firing pattern. The parameters of the action potential were determined from the first action potential evoked at a given threshold. For spontaneous firing of action potentials, the neurons were held at their resting membrane potentials with no current injection. The neurons were held at −70 mV and at 0 mV to record spontaneous excitatory postsynaptic current (sEPSC) and spontaneous inhibitory postsynaptic current (sIPSC), respectively. All recordings were performed using a multiclamp amplifier (Axon, 700B), and the signals were digitized at 10 kHz and filtered at 2 kHz using a Digital-Analog Converter named The Axon™ Digidata® 1550 Data Acquisition System (Molecular Devices, San Jose, USA).

### Statistical Analysis

The parameters of the action potential were analyzed with pClamp9. sPSCs were manually detected by the Mini Analysis Program. Values are presented as the mean ± standard error of the mean (SEM). Significant differences were assessed by the *t*-test or the Kolmogorov–Smirnov (*K–S*) test. Graphs were generated by Graphpad Prism 8.0 or Sigmaplot 12.0.

## Results

### Classification of the Neurons Into Delayed (Glutamatergic) and Tonic (GABAergic) Subtypes

**Figure 1:**
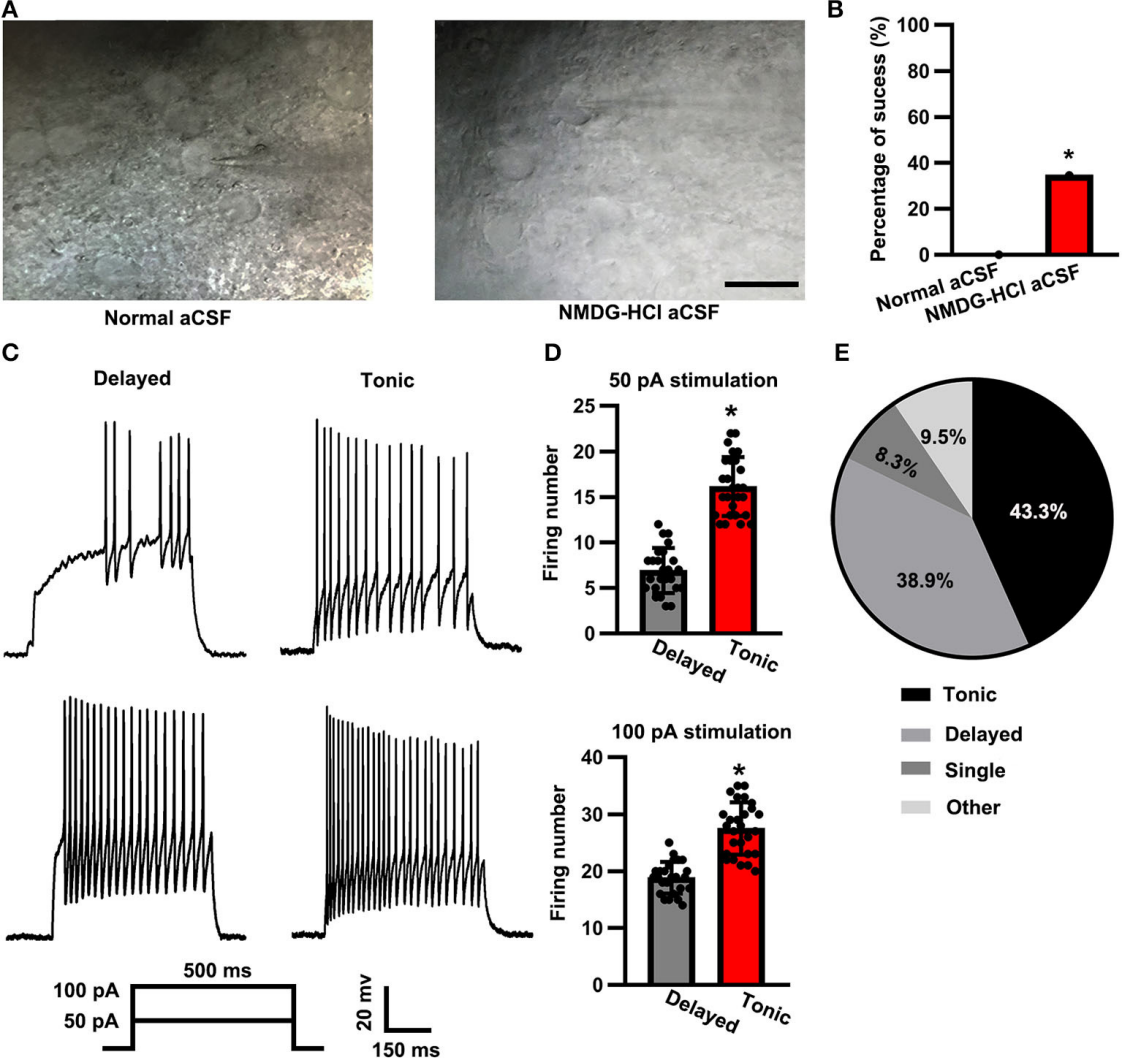
Firing patterns of substantia gelatinosa (SG) neurons. **(A)** Representative IR- differential interference contrast microscope (DIC) images of the spinal cord slices that were cut with normal artificial cerebral spinal fluid (aCSF) (left panel) vs. N-methyl-D-glucamine HCl (NMDG-HCl) aCSF (right panel). The scale bar was 20 μm. **(B)** Statistical analysis of the patch-clamp success rate between the two groups. **(C)** Representative delayed (left panel) and tonic (right panel) firing patterns at 50 and 100 pA of 500 ms current stimulation. **(D)** Statistical firing number of delayed firing pattern and tonic firing pattern neurons at stimulation of 50 pA (up panel) and 100 pA (down panel). Data were shown as mean ± standard error of the mean (SEM). **(E)** Distribution of neuron firing patterns (pie chart). *p < 0.05.

The morphological preservation of the neurons in the spinal cord slice was evaluated by the overall shape and appearance of the somata. A few neurons in the NMDG-based cutting solution group retained a bright and smooth membrane. In contrast, the neurons in the regular aCSF cutting solution group possessed a fragile membrane and an easy-to-collapse glass microelectrode pressed on the surface (Figure 1A). Based on the strict criterion described in the “Patch-clamp recording” section, we analyzed the success rate of 34.6% (90/260) in the NMDG-HCl cutting solution group (Figure 1B). However, we patched 50 neurons without a single success in the regular aCSF cutting solution group. The results concluded that the level of effectiveness of an NMDG-based aCSF cutting solution significantly increased in comparison to that of the regular aCSF cutting solution (Chi-square test, *p* < 0.01).

According to the observed firing patterns, the neurons in the SG were primarily classified into delayed and tonic subtypes (Figure 1C). Based on François et al. (2017) and Grudt and Perl (2002), the spinal dorsal horn glutamatergic neurons showed a delayed pattern, whereas the GABAergic neurons showed a tonic pattern. When stimulated with a 500-ms current pulse of 50 pA, the firing number of delayed (glutamatergic) neurons was less than that of tonic (GABAergic) neurons (delayed: 6.92 ± 2.48, *n* = 25 vs. tonic: 16.15 ± 3.22, *n* = 27; *t*-test: *p* < 0.05; Figure 1D, top panel). Similarly, the firing number of delayed (glutamatergic) neurons was also significantly less than that of tonic (GABAergic) neurons when a 100-pA current pulse was applied for a 500-ms time duration (delayed: 18.84 ± 2.79, *n* = 25 vs. tonic: 27.48 ± 4.59, *n* = 27; *t*-test: *p* < 0.05*;* Figure 1B, bottom panel). Among the 90 total recorded neurons, 43.3% (*n* = 39) displayed a tonic-firing pattern, whereas 38.9% (*n* = 35) displayed a delayed-firing pattern, 8.3% (*n* = 7) of the neurons displayed a single-firing pattern, and 9.5% (*n* = 9; Figure 1E) of the neurons were categorized as other firing patterns.

### Passive Membrane Properties, Resting Membrane Potential, and Evoked Action Potential Properties

**Figure 2:**
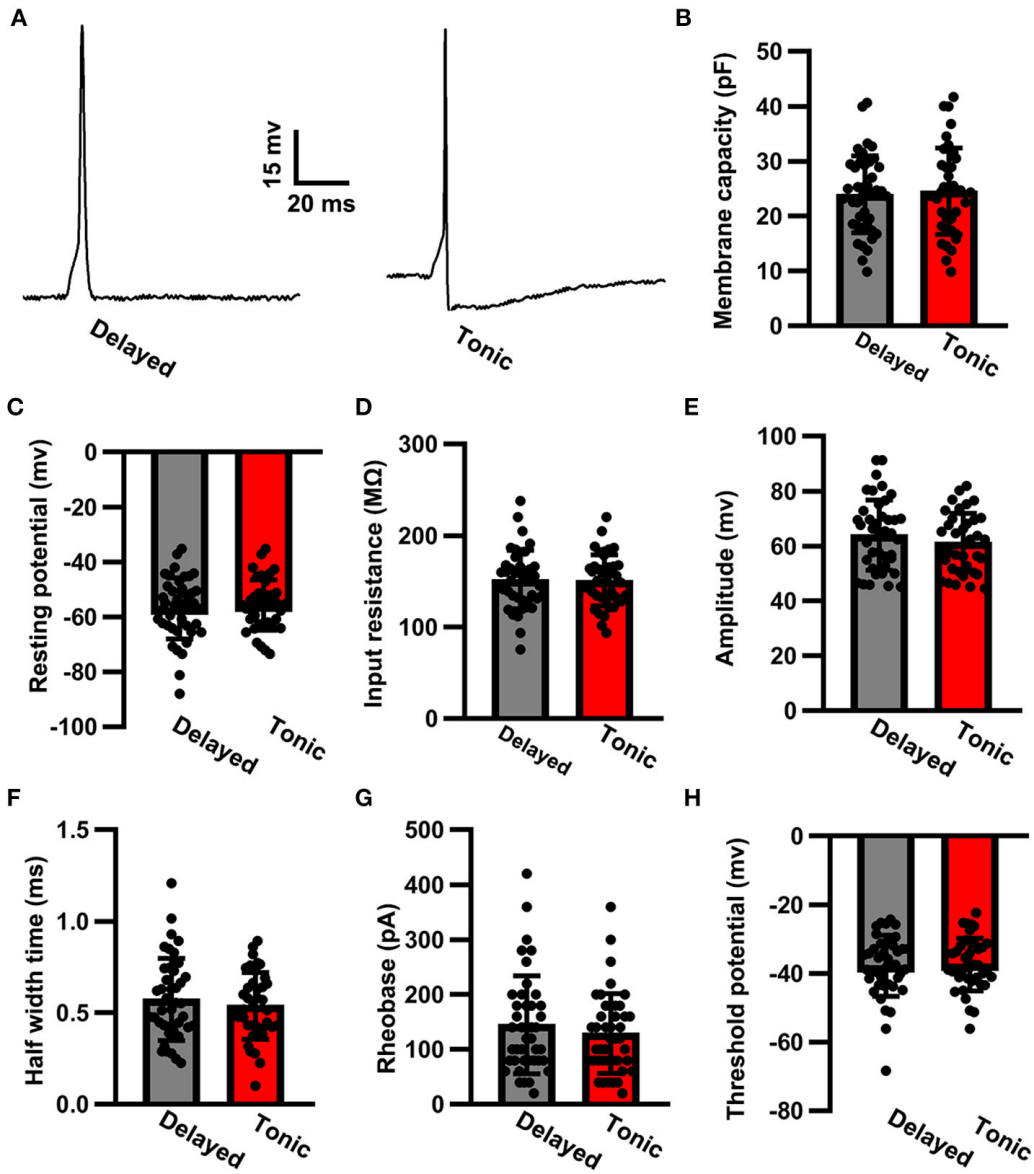
The action potential properties of SG neurons. **(A)** Examples of evoked action potential. Comparison of membrane capacity **(B)**, resting potential **(C)**, input resistance **(D)**, amplitude **(E)**, half-width time **(F)**, rheobase **(G)**, and threshold potential **(H)** of action potentials between delayed-firing pattern and tonic-firing pattern neurons. Data were shown as mean ± SEM. The time scale bar is 20 ms and the amplitude bar is 15 mv.

We injected a series of negative currents and assessed a comprehensive battery of electrophysiological properties. Between delayed (glutamatergic) and tonic (GABAergic) neurons, the input resistance was similar (delayed: 151.96 ± 32.08 MΩ, *n* = 42 vs. tonic: 151.13 ± 27.99 MΩ, *n* = 39; *t*-test: *p* > 0.05; Figure 2A). No robust differences were observed in the membrane capacity between the neuron types (delayed: 23.97 ± 7.03 pF, *n* = 42 vs. tonic: 24.56 ± 7.87 pF, *n* = 39; *t*-test: *p* > 0.05*;* Figure 2B). The resting membrane potential of delayed (glutamatergic) neurons did not differ from that of tonic (GABAergic) neurons (delayed: −56.83 ± 11.31 mV, *n* = 42 vs. tonic: −55.68 ± 9.12 mV, *n* = 39; *t*-test: *p* > 0.05*;* Figure 2C). For the evoked signal action potential (Figure 2D), no significant differences were observed in amplitude (delayed: 64.02 ± 12.79 mV, *n* = 42 vs. tonic 61.33 ± 10.64 mV, *n* = 39; *t-*test: *p* > 0.05; Figure 2E), half-width time (delayed: 0.57 ± 0.22 ms, *n* = 42 vs. tonic: 0.54 ± 0.18 ms, *n* = 39; *t*-test: *p* > 0.05; Figure 2F), rheobase (delayed: 124.39 ± 19.78 pA, *n* = 42 vs. tonic: 111.07 ± 13.75 pA, *n* = 39; *t*-test: *p* > 0.05; Figure 2G), and threshold potential (delayed: −37.92 ± 8.88 mV, *n* = 42 vs. tonic: −37.36 ± 7.67 mV, *n* = 39; *t*-test: *p* > 0.05; Figure 2H).

### sEPSC Properties

**Figure 3:**
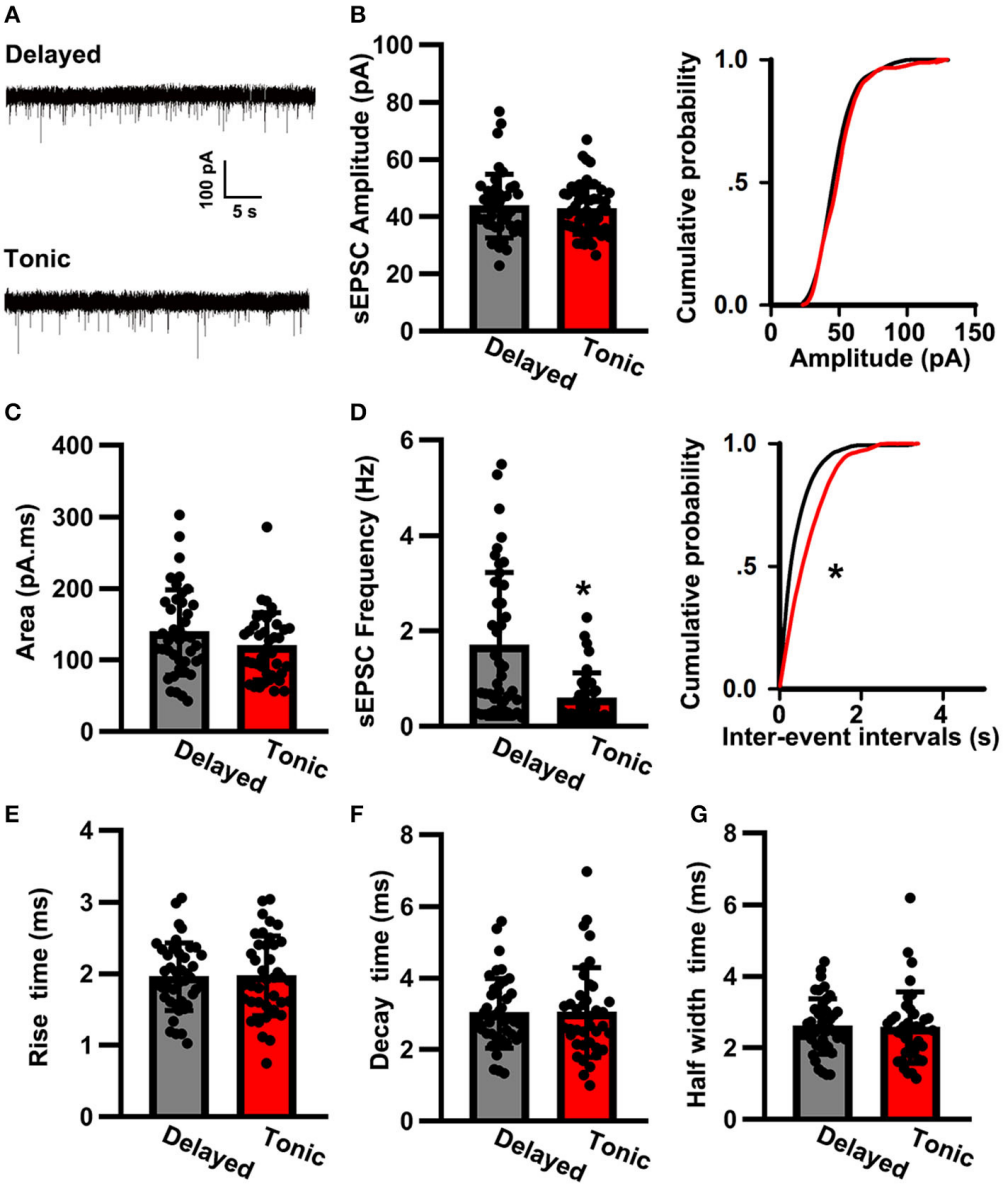
The spontaneous excitatory postsynaptic current (sEPSC) properties of SG neurons. **(A)** Examples of sEPSC in two groups. The time scale bar is 5 s and the amplitude bar is 100 pA. Comparison of amplitude **(B)**, area **(C)**, frequency **(D)**, rise time **(E)**, decay time **(F)**, and half-width time **(G)** of sEPSC between delayed-firing pattern and tonic-firing pattern neurons. Data were shown as mean ± SEM. *p < 0.05.

To further investigate the local synaptic transmission of delayed (glutamatergic) and tonic (GABAergic) neurons, we recorded sEPSCs (Figure 3A). The sEPSC amplitude of delayed (glutamatergic) neurons (43.59 ± 11.27 pA, *n* = 42) was in close approximation with that of tonic (GABAergic) neurons (41.26 ± 7.71 pA, *n* = 39; *t*-test: *p* > 0.05; Figure 3B). However, the sEPSC frequency was significantly increased in delayed (glutamatergic) neurons (1.69 ± 1.33 Hz, *n* = 42) in comparison with that of in tonic (GABAergic) neurons (0.58 ± 0.41 Hz, *n* = 39; *K–S* test, *p* < 0.05; Figure 3C). Interestingly, the two groups had convergence in area (delayed: 138.65 ± 58.64 pA.ms, *n* = 42 vs. tonic: 119.23 ± 47.07 pA.ms, *n* = 39; *t*-test: *p* > 0.05; Figure 3D). The kinetic characteristics, such as the rise time (delayed: 1.95 ± 0.46 pA.ms, *n* = 42 vs. tonic: 1.92 ± 0.53 pA.ms, *n* = 39; *t*-test: *p* > 0.05; Figure 3E), the decay time (delayed: 3.03 ± 1 ms, *n* = 42 vs. tonic: 3.18 ± 1.18 ms, *n* = 39; *t*-test*p* > 0.05; Figure 3F), and the half-width time (delayed: 2.59 ± 0.77 ms, *n* = 42 vs. tonic: 2.56 ± 0.99 ms, *n* = 39; *t*-test: *p* > 0.05; Figure 3G), were similar.

### sIPSC Properties

**Figure 4:**
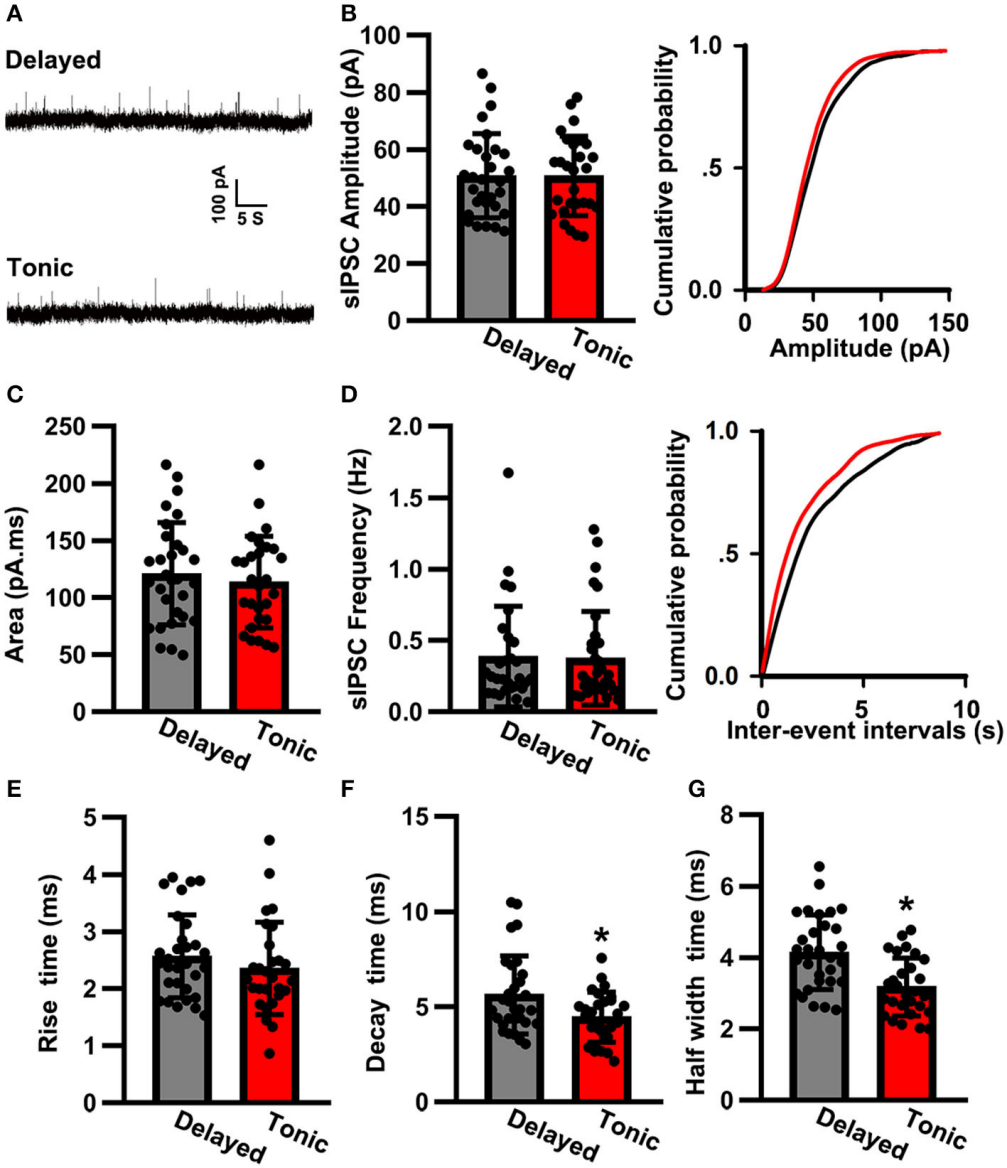
The spontaneous inhibitory postsynaptic current (sIPSC) properties of SG neurons. **(A)** Examples of sIPSC in two groups. The time scale bar is 5 s and the amplitude bar is 100 pA. Comparison of amplitude **(B)**, area **(C)**, frequency **(D)**, rise time **(E)**, decay time **(F)**, and half-width time **(G)** of sIPSC between delayed-firing pattern and tonic-firing pattern neurons. Data were shown as mean ± SEM. *p < 0.05.

We then analyzed the sIPSC properties (Figure 4A). The sIPSC amplitude of delayed (glutamatergic) neurons (50.82 ± 14.73 pA, *n* = 30) was nearly the same as that of tonic (GABAergic) neurons (50.77 ± 13.23 pA, *n* = 28; *t*-test: *p* > 0.05; Figure 4B). The two groups were also similar in terms of the area (delayed: 121.02 ± 44.81 pA.ms, *n* = 30 vs. tonic: 113.75 ± 40.12 pA.ms, *n* = 28; *t*-test: *p* > 0.05; Figure 4C) and frequency of sIPSCs (delayed: 0.38 ± 0.31 Hz, *n* = 30 vs. tonic: 0.36 ± 0.30 Hz, *n* = 28; *K–S* test*: p* > 0.05; Figure 4D). Although the rise time of delayed neurons (2.56 ± 0.73 ms, *n* = 30) was similar to that of tonic neurons (2.36 ± 0.74 ms, *n* = 28; *t*-test: *p* > 0.05; Figure 4E), significant differences were observed in the decay times (delayed: 5.63 ± 2.02 ms, *n* = 30 vs. tonic: 4.46 ± 1.31 ms, *n* = 28; *t-*test: *p* < 0.05; Figure 4F) and half-width times (delayed: 4.15 ± 1.03 ms, *n* = 30 vs. tonic: 3.27 ± 0.81 ms, *n* = 28; *t*-test: *p* < 0.05; Figure 4G).

## Discussion

So far, several attempts have been made to perform the electrophysiological recordings from the neurons in the preparation of the spinal cord slices made from adult animals. Geoffrey et al. described the preparation of the spinal cord slices from adult rats aging 3–6 weeks and applied extracellular recordings to study the spontaneous and afferent-evoked firing properties (Bentley and Gent, 1994). However, we disagree with the classification of animals younger than 8 weeks as adults. Pratip et al. classified animals older than 6 weeks of age (P42 and above) as adults for the purposes of recording from lumbar motoneurons (Mitra and Brownstone, 2012). However, we were unable to obtain satisfactory information regarding the age of the experimental rodents used, which may have been either 8 or 16 weeks. In Yoshimur's research, 8–16-week-old Sprague Dawley rats were sacrificed to prepare the spinal cord slices (Yoshimura and Nishi, 1993). Even at 16 weeks old, rats can only be identified as socially mature. Ataka demonstrated that baclofen inhibits the glutamatergic transmission evoked by C-afferents more effectively than those evoked by Aδ-afferents using whole-cell patch-clamp recordings on the spinal cord slices obtained from 7–8-week-old Sprague Dawley rats (Ataka et al., 2000). In brief, most previous preparations of the spinal cord slices mainly have primarily focused on young adult rodents. In recent years, the NMDG-substituted aCSF recovery method has been applied for the preparation of brain slices spanning a wide range of animal ages and has been demonstrated to reliably protect the neurons in the obtained slices. Although NMDG aCSF has been used during the preparation of the spinal cord slices for the performance of the patch-clamp recordings on the neurons obtained from juvenile Sprague Dawley rats (3–5 weeks) (Zhu et al., 2019a) or white matter glia of young adult female Wistar rats (>2 months, 240–260 g) (Nashmi et al., 2002), it remains unknown whether this method was suitable for the preservation of the spinal cord slices obtained from mature, older rodents for targeting neuron recording. To our knowledge, we are the first group to perform the patch-clamp recordings on the neurons from the spinal cord slice of middle-aged rats prepared by NMDG-HCl aCSF rather than the regular aCSF cutting solution. In comparison with that of juvenile or young adult rodent, the tissue slice of aging rodent has a low resilience, so we speculated that high concentrations of sodium in the regular aCSF might not have effectively protected and revitalized the neurons that are subjected to the trauma of the slicing procedure. Furthermore, we agreed with the theory that NMDG-HCl-based aCSF decrease the passive influx of Na^+^ and thus alleviate neuronal edema through water entry. In addition, ascorbic acid and sodium pyruvate were supplied to serve as powerful antioxidants. Meanwhile, N-acetyl-L-cysteine (NAC) was also proposed to maintain the intracellular glutathione level and scavenge oxygen-free radicals and peroxides. These appropriate modifications would contribute to the routine preparation of middle-aged rodent spinal cord slices for various applications.

Whole-cell patch-clamp recordings from neurons in the spinal cord slices obtained from young adult rodents, in combination with immunohistochemistry and morphological analyses, have resulted in the extensive study of lamina II neurons. A vast majority of neurons in laminae II can be divided into two primary classes: excitatory and inhibitory. Toshiharu found that in lamina II of the rat spinal cord, 50.75% (34/67) of the neurons were vesicular glutamate transporter (VGLUT2) immunoreactive and were identified as excitatory neurons, whereas the remaining 49.25% (33/67) of the neurons were vesicular GABA transporter (VGAT) immunoreactive and identified as inhibitory neurons (Yasaka et al., 2010). Furthermore, their electrophysiological results showed that all GABAergic neurons showed a tonic firing pattern, whereas 81.81% (18/22) of the glutamatergic neurons showed a delayed or transient firing pattern. In contrast, Santos reported that 85.29% (87/102) of SG interneurons were excitatory and glutamatergic, whereas the remaining 14.71% (15/102) of the neurons were inhibitory (Santos et al., 2007). In our opinion, the significant differences between these studies are likely due to methodological differences, such as differences in the criteria used for valuation, the procedures used for slice preparation, and the random selection of the targeted neurons. Increasingly, transgenic mouse models have been used for extensive studies, including the study of electrophysiological characteristics of spinal cords featuring specifically labeled GABAergic and glutamatergic neurons. Labrakakis reported that 84.38% (27/32) of GAD65::eGFP neurons (featuring a green fluorescent protein-labeled glutamic acid decarboxylase 65-kilodalton isoform) in lamina II of the spinal cord showed a tonic-firing pattern (Labrakakis et al., 2009). Consistently, Punnakkal confirmed that 91.3% (21/23) of GAD67::eGFP neurons exhibited a tonic firing pattern. We concluded that neuronal firing patterns are expected to be closely related to neuronal types, based on the findings of previous studies, and in our study, we classified the neurons according to their firing patterns. Using this method, we found that 43.3% (39/90) of the neurons were tonic (GABAergic), which was a slightly smaller proportion than those classified as glutamatergic. Compared with younger rodents, the neuroarchitecture of the SG area in middle-aged rodents appeared to be similar. These results suggested that a neuronal-type differentiation is likely to be suspended during the adolescence period; however, the occurrence of changes to neuronal circuits or the activity remains unclear.

In Punnakkal's research, the resting potential, membrane capacitance, and input resistance of GABAergic neurons were similar to those of glutamatergic neurons. In contrast, GABAergic neurons had a lower rheobase and more hyperpolarized potential thresholds, indicating that they required less excitatory inputs for activation. The action potentials of GAD67::eGFP neurons were longer than those of vGLUT2::eGFP neurons (Punnakkal et al., 2014). In contrast, we found no significant differences in the passive membrane properties and evoked signal action potential properties between delayed (glutamatergic) and tonic (GABAergic) neurons in the SG of the spinal dorsal horn obtained from middle-aged rats. These results indicated that the electrophysiological properties of the neurons might converge with aging. To the best of our abilities, we were unable to identify any published studies that compared sEPSCs or sIPSCs between glutamatergic and GABAergic neurons, but only the studies that examined all neurons in the dorsal horn. In Wang's research, the frequency of sEPSCs in spinal dorsal horn neurons was 7.1 ± 2.9 Hz. Compared with sEPSCs, sIPSCs presented a lower frequency of 4.4 ± 0.4 Hz. However, the amplitude of sEPSCs did not differ from that of sIPSCs (10 ± 1.2 pA) (Wang et al., 2017). These results differed from those reported by Chong, who recorded all SG neurons from a young adult rat. We inferred that the difference in the preparation of the slice and patch recording systems would be accounted for. In our study, we recorded sEPSCs and sIPSCs on the same neuron by clamping at −70 mV and 0 mV, respectively, without inhibitors. Yet, it must be noticed that there was a chance to record sIPSC during sEPSC recordings at Vh of −70 mV in our recording method. Meanwhile, the recorded sIPSC might be contaminated by the sEPSC component from NMDA and GluA2-lacking AMPA receptors. As reversal potential could not completely separate sIPSC and sEPSC, we recommend that bicuculline should be used to inhibit GABA_A_ receptors for an appropriate recording of sEPSC; in turn, AMPA receptor antagonist NBQX (CNQX) and NMDA receptor antagonist D-APV should be presented to block glutamatergic synaptic transmission for the strict recording of sIPSC. We found that the frequency of sEPSCs in delayed (glutamatergic) neurons was higher than that for tonic (GABAergic) neurons. These results suggested that glutamatergic neurons may receive more excitatory synaptic transmissions than GABAergic neurons. The decay time for sIPSCs in delayed (glutamatergic) neurons was longer than that for tonic (GABAergic) neurons, indicating an increased quantal release from presynaptic neurons onto glutamatergic neurons.

In summary, we successfully applied the NMDG-HCl solution for the preparation of the spinal cord slice from middle-aged rodents and performed the patch-clamp recordings on the neurons in the lamina II layer of the spinal cord. According to the action potential firing patterns, the neurons were classified into delayed (glutamatergic) and tonic (GABAergic) subtypes. The sEPSC frequency of delayed (glutamatergic) neurons was higher than that of tonic (GABAergic) neurons, and the sIPSC decay time for delayed (glutamatergic) neurons was longer than that of tonic (GABAergic) neurons. We anticipate that, in future studies, spinal cord slice recordings of middle-aged rodents, in combination with optogenetics, transgenic, and chemogenetics, can be used to detect changes in neuronal properties associated with the older period of life and aging-associated diseases.

## Data Availability Statement

The raw data supporting the conclusions of this article will be made available by the authors, without undue reservation.

## Ethics Statement

The animal study was reviewed and approved by Institute of Laboratory Animal Science, Hubei University of Medicine.

## Author Contributions

CK: conceptualization, writing—review, editing, and supervision. YL, SS, and JY: investigation. MP and SW: formal analysis. YL and SS: writing—original draft. YL, SS, and CK: funding acquisition. All authors contributed to the article and approved the submitted version.

## Conflict of Interest

The authors declare that the research was conducted in the absence of any commercial or financial relationships that could be construed as a potential conflict of interest.
